# Predictability of Extreme Events in Social Media

**DOI:** 10.1371/journal.pone.0111506

**Published:** 2014-11-04

**Authors:** José M. Miotto, Eduardo G. Altmann

**Affiliations:** Max Planck Institute for the Physics of Complex Systems, Dresden, Germany; University of Warwick, United Kingdom

## Abstract

It is part of our daily social-media experience that seemingly ordinary items (videos, news, publications, etc.) unexpectedly gain an enormous amount of attention. Here we investigate how unexpected these extreme events are. We propose a method that, given some information on the items, quantifies the predictability of events, i.e., the potential of identifying in advance the most successful items. Applying this method to different data, ranging from views in YouTube videos to posts in Usenet discussion groups, we invariantly find that the predictability increases for the most extreme events. This indicates that, despite the inherently stochastic collective dynamics of users, efficient prediction is possible for the most successful items.

## Introduction

When items produced in social media are abundant, the public attention is the scarce factor for which they compete [1]–[3]. Success in such *economy of attention* is very uneven: the distribution of attention across different items typically shows heavy tails which resemble Pareto's distribution of income [4] and, more generally, are an outcome of complex collective dynamics [5]–[12] and non-trivial maximizations of entropic functions [13], [14]. Increasing availability of large databases confirm the universality of these observations and renew the interest on understanding the dynamics of attention, see Tab. 1.

**Table 1:** Examples in which fat-tailed distributions of popularity across items have been reported.

System	Item	Attention measure	Refs.
Online Videos	video	views, likes	[18]
Discussion Groups	threads	posts, answers	[38]
Publications	papers	citations, views	[6], [8], [15], [19]
Twitter	tweet	retweets	[9]
WWW	webpage	views	[11]
Online Petitions	petition	signers	[39]

**Figure 1:**
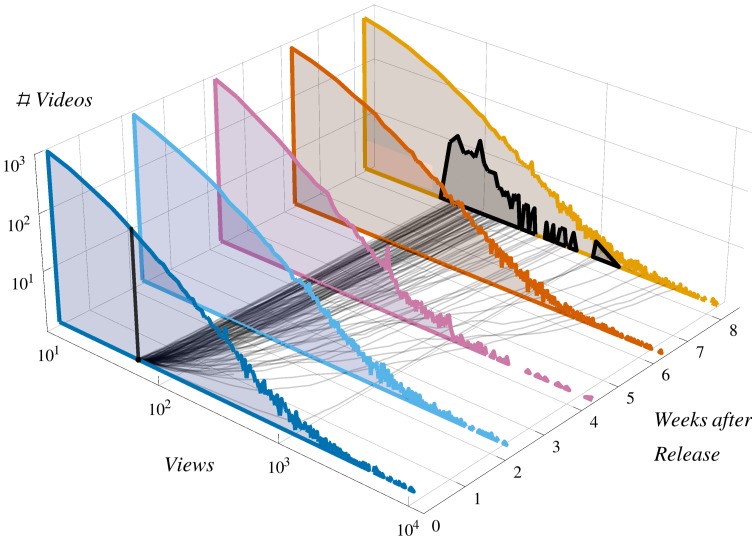
Dynamics of views in YouTube. **Colored histograms**: distributions of views at fixed times after publication (0.3 million videos from our database). **Gray lines at the bottom**: trajectories of 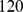 videos which had the same early success (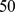 views 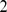 days after publication). **Black histogram**: distribution of views of the 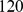 selected videos 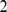 months after publication.

Universal features of heavy-tailed distributions do not easily lead to a good forecast of specific items [5], a problem of major fundamental and practical interest [15]–[19]. This is illustrated in Fig. 1, which shows that the heavy-tailed distribution appears at very short times but items with the same early success have radically different future evolutions. The path of each item is sensitively dependent on idiosyncratic decisions which may be amplified through collective phenomena. An important question is how to quantify the extent into which prediction of individual items is possible (i.e., their *predictability*) [20]. Of particular interest –in social and natural systems– is the predictability of extreme events [21]–[26], the small number of items in the tail of the distribution that gather a substantial portion of the public attention.

Measuring predictability is difficult because it is usually impossible to disentangle how multiple factors affect the quality of predictions. For instance, predictions of the attention that individual items are going to receive rely on (i) information on properties of the item (e.g., metadata or the attention received in the first days) and (ii) a prediction strategy that converts the information into predictions. The quality of the predictions reflect the interplay between these two factors and the dynamics of attention in the system. In particular, the choice of the prediction strategy is crucial. Instead, predictability is a property of the system and is by definition independent of the prediction strategy (it is the upper bound for the quality of any prediction based on the same information on the items). A proper measure of the predictability should provide direct access to the properties of the system, enabling a quantification of the importance of different information on the items in terms of their predictive power.

In this paper we introduce a method to quantify the predictability of extreme events and apply it to data from social media. This is done by formulating a simple prediction problem which allows for the computation of the optimal prediction strategy. The problem we consider is to provide a binary (yes/no) prediction whether an item will be an extreme event or not (attention passes a given threshold). Predictability is then quantified as the quality of the optimal strategy. We apply this method to four different systems: views of YouTube videos, comments in threads of Usenet discussion groups, votes to Stack-Overflow questions, and number of views of papers published in the journal PLOS ONE. Our most striking empirical finding is that in all cases the predictability increases for more extreme events (increasing threshold). We show that this observation is a direct consequence of differences in (the tails of) the distributions of attention conditioned by the known property about the items.

The paper is divided as follows: Sec. Motivation motivates the problem of event prediction by showing that it is robust to data with heavy tails. Sec. Methods introduces the method to quantify predictability, which is used in the Sec. Application to Data. A summary of our findings appears in Sec. Conclusions.

## Motivation

### Characterization of Heavy-tails

Different systems in which competition for attention takes place share similar statistical properties. Here we quantify attention of published items in 4 representative systems (see Appendix S1, Sec. 1 for details; all the data is available in Ref. [27]):

- views received by 16.2 million videos in YouTube.com between Jan. 2012 and Apr. 2013;
- posts written in 0.8 million threads in 9 different Usenet discussion groups between 1994 and 2008;
- votes to 4.6 million questions published in Stack-Overflow between Jul. 2008 and Mar. 2013.
- views of 72246 papers published in the journal PLOS ONE from Dec. 2006 to Aug. 2013 (see also Ref. [28]).

The tails of the distribution 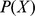 of attention 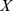 (views, posts, etc.) received by the items (videos, threads, etc.) at a large time 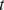 after publication is characterized without loss of generality using Extreme Value Theory. It states that for large thresholds 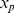 the probability 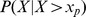 follows a Generalized Pareto distribution [29]

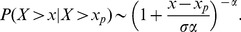
(1)


The fits of different partitions of our databases yield 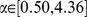 and are statistically significant already for relatively small 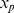
's (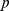-value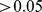 in 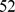 out of 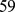 fits, see Appendix S1, Sec. 2 and Fig. S1 for details). These results confirm the presence of heavy tails, an observation reported previously in a variety of cases (see Tab. 1). This suggests that our databases are representative of social media more generally (while scientific publications are usually not classified as social media items, from the point of view of their online views, they are subject to the same attention-gathering process).

### Prediction of Extreme Events

Prediction in data with heavy tails is typically not robust. As an example, consider using as a predictor 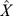 of the future attention the mean 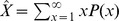, which is the optimal predictor, if we measure the quality of prediction with the standard deviation of 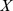. For heavy-tailed distributions, the mean and standard deviation may not be defined (for 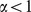 and 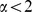, respectively), making prediction not robust (i.e., it depends sensitively on the training and target datasets). This illustrates the problems heavy-tails typically appear when value predictions are issued and indicates the need for a different approach to prediction of attention.

We consider the problem of *event prediction* because, as shown below, it is robust against fat-tailed distributions. We say an event 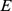 happens at time 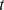 if the cumulative attention 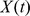 received by the considered item until time 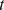 is within a given range of values. We are particularly interested in predicting extreme events 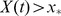, i.e., to determine whether the attention to an item passes a threshold 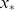 before time 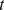. The variable to be predicted for each item is binary: 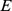 or 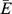 (not 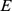). We consider the problem of issuing binary predictions for each item (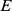 will occur or not), which is equivalent to a classification problem and different from a probabilistic prediction (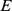 will occur with a given probability). Heavy tails do not affect the robusteness of the method because all items for which 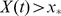 count the same (each of them as one event), regardless of their size 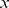. Indeed, the tails of 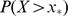 determine simply how the probability of an event 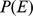 depends on the threshold 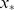 (we assume 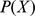 exists).

## Methods

In this section we introduce a method to quantify predictability based on the binary prediction of extreme events. This is done by arguing that, despite the seeming freedom to choose between different prediction strategies, it is possible to compute a single optimal strategy for this problem. We then show how the quality of prediction can be quantified and argue that the quality of the optimal strategy is a proper quantification of predictability.

Predictions are based on information on items which generally lead to a partition of the items in groups 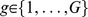 that have the same feature [30]. As a simple example of our general approach, consider the problem of predicting at publication time 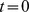 the YouTube videos that at 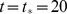 days will have more than 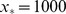 views (about 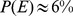 of all videos succeed). As items' information, we use the category of a video so that, e.g., videos belonging to the category *music* correspond to one group 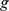 and videos belonging to *sport* correspond to a different group 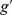. Since the membership to a group 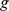 is the only thing that characterizes an item, predictive strategies can only be based on the probability of having 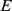 for that group, 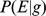.

In principle, one can think about different strategies on how to issue binary predictions on the items of a group 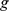. They can be based on the likelihood (L) 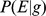 or on the posterior (P) probability 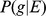 [22], and they can issue predictions stochastically (S), with rates proportional to the computed probabilities, or deterministically (D), only for the groups with largest 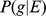 or 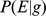. These simple considerations lead to four (out of many) alternative strategies to predict events (raise alarms) for items in group 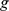


**(LS)** stochastically based on the likelihood, i.e., with probability 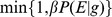, with 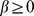;

**(LD)** deterministically based on the likelihood, i.e., always if 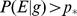, with 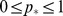;

**(PS)** stochastically based on the posterior, i.e., with probability 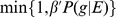, with 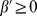;

**(PD)** deterministically based on the posterior, i.e., always if 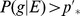, with 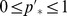.

In the limit of large number of predictions (items), the fraction of events that strategy (LS) predicts for each group 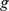 matches the probability of events 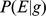 and therefore strategy (LS) is *reliable* [31] and can be considered a natural extension of a probabilistic predictor. Predictions of strategies (LD), (PS) and (PD) do not follow 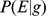 and therefore they are not reliable.

**Figure 2:**
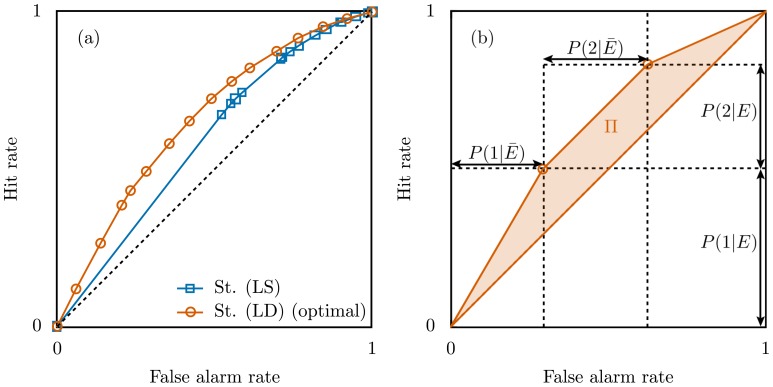
Quantifying the quality of event-prediction strategies requires measuring both the hit and false alarm rates. (**a**) Performance of Strategy (LS) and Strategy (LD) for the problem of predicting views of YouTube videos 20 days after publication based on their categories. The symbols indicate where the rate of issued predictions for a given group equals 1 (the straight lines between the symbols are obtained by issuing predictions randomly with a growing rate). (**b**) Illustration of the prediction curve (red line) for an optimal strategy with three groups 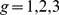 with 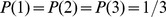 and 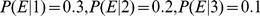.

The quality of a strategy for event prediction is assessed by computing the false alarm rate (or False Positive Rate, equal to one minus the specificity) and the hit rate (True Positive Rate, equal to the sensitivity) over all predictions (items), see Appendix S1, Sec. 3 for details. Varying the amount of desidered false alarms of the prediction strategy (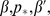 and 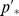 in the examples above), a curve in the hit 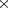 false-alarm space is obtained, see Fig. 2(a). The overall quality is measured by the area below this curve, known as Area Under the Curve (AUC) [32]. For convenience, we use the area between the curve and the diagonal (hits = false-alarms), 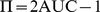 (equivalent to the Gini coefficient). In this way, 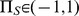 represents the improvement of strategy 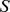 against a random prediction. In absence of information 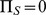 and perfect predictions lead to 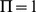. In the YouTube example considered above, we obtain 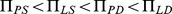 (17%, 18%, 29%, 32%), indicating that strategy (LD) is the best one.

We now argue that strategy (LD) is optimal (or *dominant* [33]), i.e., for any false alarm rate it leads to a larger hit rate than any other strategy based on the same set of 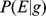. To see this, notice that strategy (LD) leads to a piecewise linear curve, see Fig. 2(b), and is the only ordering of the groups that enforces convexity in the hit 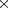 false-alarms rates space, see Appendix S1, Sec. 4 for a formal derivation. The ranking of the groups by 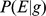 implies a ranking of the items, an implicit assumption in the measure of the performance of classification rules [32], [34]. The existence of an optimal strategy implies that the freedom in choosing the prediction strategy argued above is not genuine and that we can ignore the alternative strategies. In our context, it implies that the performance of the optimal strategy measures a property of the system (or problem), and not simply the efficiency of a particular strategy. Therefore, we use the quality of prediction of the optimal strategy (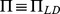) to quantify the predictability (i.e., the potential prediction) of the system for the given problem and information. By geometrical arguments we obtain from Fig. 2 (b) (see Appendix S1, Sec. 5) 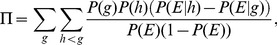
(2)where 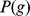 is the probability of group 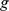 and 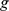 is ordered by decreasing 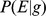, i.e., 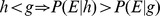.

The value of 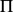 can be interpreted as the probability of a correct classification of a pair of 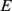 and 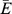 items [32], [34]. In practice, the optimality of this strategy is dependent on the estimation of the ordering of the groups according to 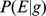. Wrong ordering may occur due to finite sampling on the training dataset or non-stationarities in the data. In fact, any permutation of indexes in Eq. (2) reduces 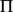.

## Results

### Application to Data

Here we apply our methodology to the four social-media data described above. We consider the problem of predicting at time 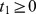 whether the attention 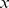 of an item at time 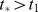 will pass a threshold 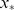. In practice, the calculation of 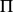 from the data is done counting the number of items: (i) in each group 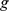 [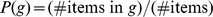]; (ii) that lead to an event 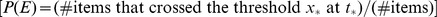; and (iii) that lead to an event given that they are in group 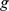 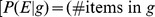 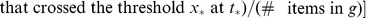. Finally, the groups are numbered as 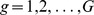 by decreasing 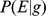 and the sum over all groups is computed as indicated in Eq. (2). In Ref. [27] we provide a python script which performs this calculation in the data.

We report the values of 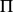 obtained from Eq. (2) considering two different informations on the items:

1. the attention at prediction time 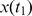;
2. information available at publication time 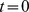 (metadata).

In case 1), a group 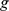 corresponds to items with the same 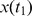. These groups are naturally ordered in terms of 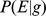 by the value of 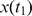 and therefore the optimal strategy is equivalent to issue positive prediction to the items with 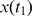 above a certain threshold. In case 2), the groups correspond to items having the same meta-data (e.g., belonging to the same category). In this case, we order the groups according to the empirically observed 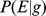 (as discussed above). Before performing a systematic exploration of parameters, we illustrate our approach in two examples:

- Consider the case of predicting whether YouTube videos at 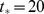 days will have more than 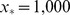 views. For case 1), we use the views achieved by the items after 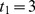 days and obtain a predictability of 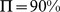. For case 2), we obtain that using the day of the week to group the items leads to 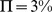 against 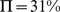 obtained using the categories of the videos. This observation, which is robust against variations of 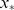 and 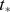, shows that the category but not the day of the week is a relevant information in determining the occurrence of extreme events in YouTube.
- Consider the problem of identifying in advance the papers published in the online journal PLOS ONE that received at least 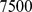 views 2 years after publication, i.e 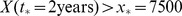 (only 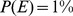 achieve this threshold). For case 1), knowing the number of views at 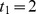 months after publication leads to a predictability of 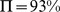. For case 2), a predictability 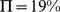 is achieved alone by knowing the number of authors of the paper –surprisingly, the chance of achieving a large number of views decays monotonously with number of author (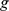 increases with number of authors).

**Figure 3:**
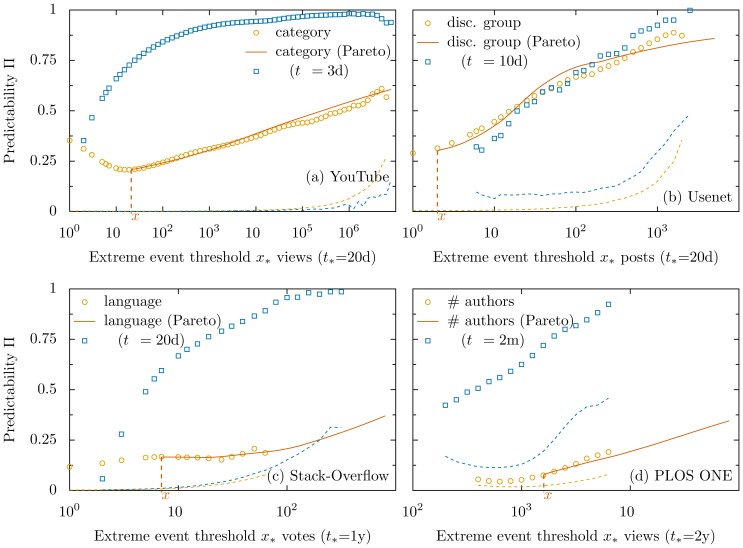
Predictability increases for extreme events. If the attention an item receives at time 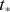 is above a threshold, 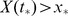, an event 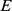 is triggered. The plots show how the predictability 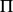 changes with 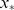 using two different informations to combine the items in groups 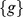. **Black circles**: 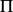 at time 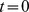 using metadata of the items to group them. The **red lines** are computed using as probabilities 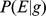 the Extreme Value distribution fits for each group at a threshold value 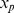, see Eq. (1) and SI Sec. 2. **Blue squares**: 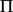 at time 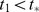 using 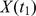, i.e., the attention the item obtained at day 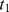. The **dashed lines** are the values of the 95% percentile of the distribution generated by measuring 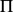 in an ensemble of databases obtained shuffling the attribution of groups (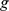) to items (the colors match the symbols and symbols are shown only where 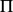 is at least twice this value). Results for the four databases are shown: (**a**) YouTube (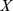: views of a video; metadata: video category); (**b**) Usenet discussion groups (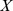: posts in a thread; metadata: discussion group of the thread); (**c**) Stack-Overflow (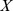: votes to a question; metadata: programming language of the question, see SI Sec. 2 for details); (**d**) PLOS ONE (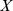: online views of a paper; metadata: number of authors of the paper).

The examples above show that formula (2) allows for a quantification of the importance of different factors (e.g., number of authors, early views to the paper) to the occurrence of extreme events, beyond correlation and regression methods (see also Ref. [19]). Besides the quantification of the predictability of specific problems, by systematically varying 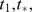 and 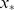 we can quantify how the predictability changes with time and with event magnitude. Our most significant finding is that in all tested databases and grouping strategies the predictability increases with 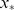, i.e., extreme events become increasingly more predictable, as shown in Fig. 3.

## Discussion

We now explain why predictability increases for extreme events (increasing 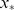). We first show that this is not due to the reduction of the number of events 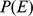. Consider the case in which 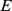 is defined in the interval 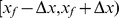. Assuming 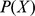 to be smooth in 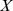, for 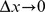 at fixed 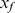 we have that 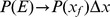 and 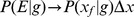 (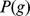 remains unaffected), and Eq. (2) yields 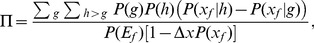
(3)which decreases with 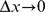. This shows that the increased predictability with 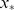 is not a trivial consequence of the reduction of 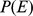 (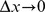), but instead is a consequence of the change in 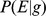 for extreme events 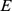.

Systematic differences in the tails of 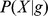 lead to an increased predictability of extreme events. Consider the case of two groups with cumulative distributions 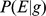 that decay as a power law as in Eq. (1) with exponents 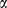 and 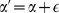, with 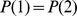. From Eq. (2), 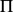 for large 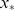 (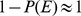) can be estimated as 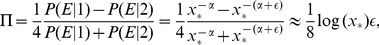
(4)where the approximation corresponds to the first order Taylor expansion around 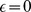. The calculation above can be directly applied to the results we obtained issuing predictions based on metadata. The logarithmic dependency in Eq. (4) is consistent with the roughly linear behavior observed in Fig. 3(a,b). A more accurate estimation is obtained using the power-law fits of Eq. (1) for each group 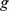 and introducing the 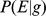 obtained from these fits in Eq. (2). The red line in Fig. 3 shows that this estimation agrees with the observations for values 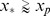, the threshold used in the fit. Deviations observed for 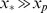 (e.g., for PLOS ONE data in panel (d)) reflect the deviations of 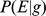 from the Pareto distribution obtained for small thresholds 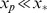. This allows for an estimation of the predictability for large thresholds 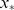 even in small datasets (when the sampling of 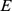 is low).

A similar behavior is expected when prediction is performed based on the attention obtained at short times 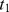. Eq. (3) applies in this case too and therefore the increase in predictability is also due to change in 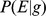 with 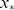 for different 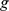 (and not, e.g., due to the decrease of 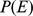). For increasingly large 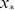 the items with significant probability of passing threshold concentrate on the large 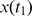 and increase the predictability of the system. We have verified that this happens already for simple multiplicative stochastic processes, such as the geometric Brownian motion (see Fig. S2). This provides further support for the generality of our finding. The dynamics of attention in specific systems affect the shape of predictability growth with threshold.

Altogether, we conclude that the difference in (the tails of) the distribution of attention of different groups 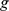 is responsible for the increase in predictability for extreme events: for large 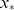, any informative property on the items increases the relative difference among the 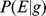. This corresponds to an increase of the information contained in the grouping which leads to an increase in 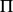.

## Conclusions

In summary, we propose a method, Eq. (2), to measure the predictability of extreme events for any given available information on the items. We applied this measure to four different social media databases and quantified how predictable the attention devoted to different items is and how informative are different properties of the items. We quantified the predictability due to metadata available at publication date and due to the early success of the items and found that usually the latter quickly becomes more relevant than the former. Our results can also be applied for combinations of different informations on the items (e.g., a group 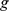 can be composed by videos in the category *music* with a fixed 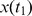). In practice, the number of groups 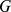 should be much smaller than the observations in the training dataset to ensure an accurate estimation of 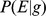. Our most striking finding is that extreme events are better predictable than non-extreme events, a result previously observed in physical systems [23] and in time-series models [22], [26]. For social media, this finding means that for the large attention catchers the surprise is reduced and the possibilities to discriminate success enhanced.

These results are particularly important in view of the widespread observation of fat-tailed distributions of attention, which imply that extreme events carry a significant portion of the total public attention. Similar distributions appear in financial markets, in which case our methodology can quantify the increase in predictability due to the availability of specific information (e.g., in Ref. [35] Internet activities were used as information to issue predictions). For the numerous models of collective behavior leading to fat tails [6], [8]–[11], [15], [19], the predictability we estimate is a bound to the quality of binary event predictions. Furthermore, our identifications of the factors leading to an improved predictability indicate which properties should be included in the models and which ones can be safely ignored (feature selection). For instance, the relevant factors identified in our analysis should affect the growth rate of items in rich-get-richer models [11], [12] or the transmission rates between agents in information-spreading models [36]. The use of 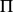 to identify relevant factors goes beyond simple correlation tests and can be considered as a measure of causality in the sense of Granger [37].

Predictability in systems showing fat tails has been a matter of intense debate. While simple models of self-organized criticality suggest that prediction of individual events is impossible [5], the existence of predictable mechanisms for the very extreme events has been advocated in different systems [24]. In practice, predictability is not an yes/no question [7], [20] and the main contribution of this paper is to provide a robust quantification of the predictability of extreme events in systems showing fat-tailed distributions.

## Supporting Information

Figure S1 **Distribution functions for each dataset.** Dashed red line: fit of the generalized Pareto distribution (see Appendix S1 Sec. 2); Gray lines: each of the categories (see Appendix S1 Sec. 2); Blue solid line: combined data.
(PDF)

Figure S2
**Predictability of simple stochastic processes.** An ensemble of random walkers evolve through the dynamics 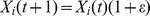, where 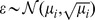 (Geometric Brownian Motion with Gaussian steps. The predictability of extreme events 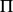 was computed for 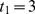 steps and 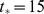 steps. GBM: 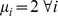 and 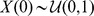; GBM heterogeneous: 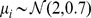 and 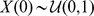, fixed in time; GBM, init exp: 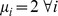 and 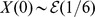; GBM, 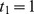 the same as GBM for 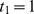; GBM, time decay: model proposed in Ref. [15], similar to GBM heterogeneous but with a rate that decays in time (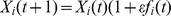 with 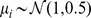; 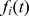 is a log-normal surviving probability with parameters 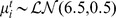 and 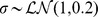).(PDF)

Appendix S1 **Details on procedures, analysis and data.**
(PDF)
