# Supplementary figures and images for: Predictability of Extreme Events in Social Media
Source: PLoS One. 2014 Nov 4;9(11):e111506. doi: 10.1371/journal.pone.0111506 (PMC4219754; doi:10.1371/journal.pone.0111506)

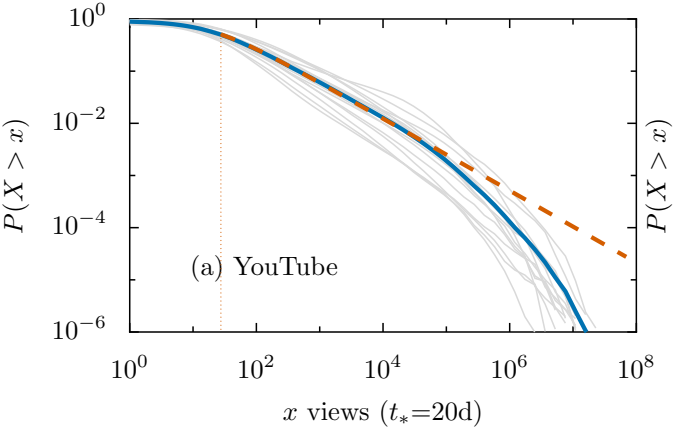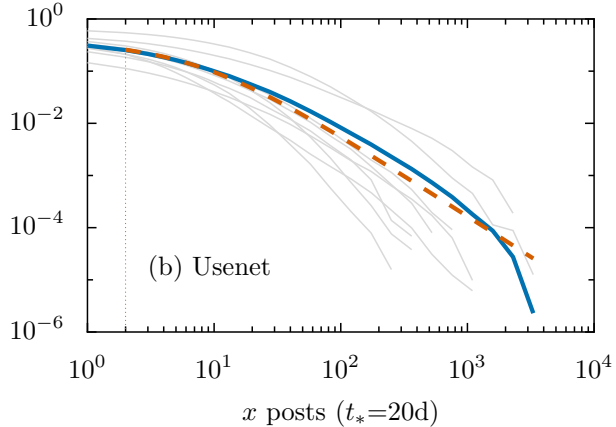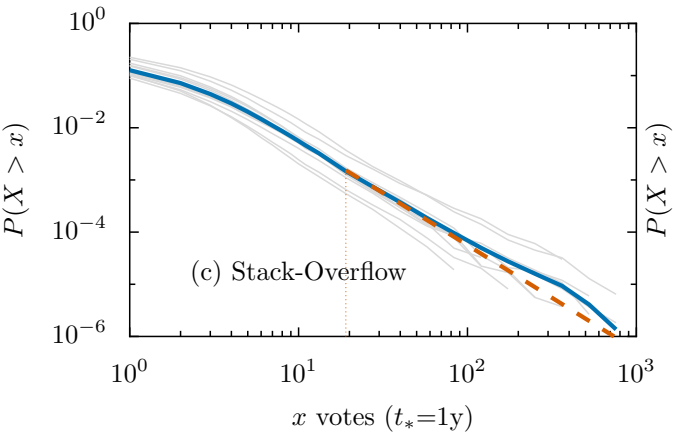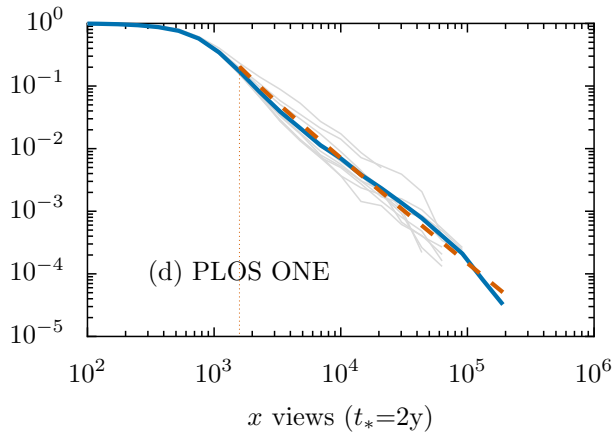

Supplement: Figure S1 — Distribution functions for each dataset. Dashed red line: fit of the generalized Pareto distribution (see Appendix S1 Sec. 2); Gray lines: each of the categories (see Appendix S1 Sec. 2); Blue solid line: combined data. (PDF) [file pone.0111506.s001.pdf]

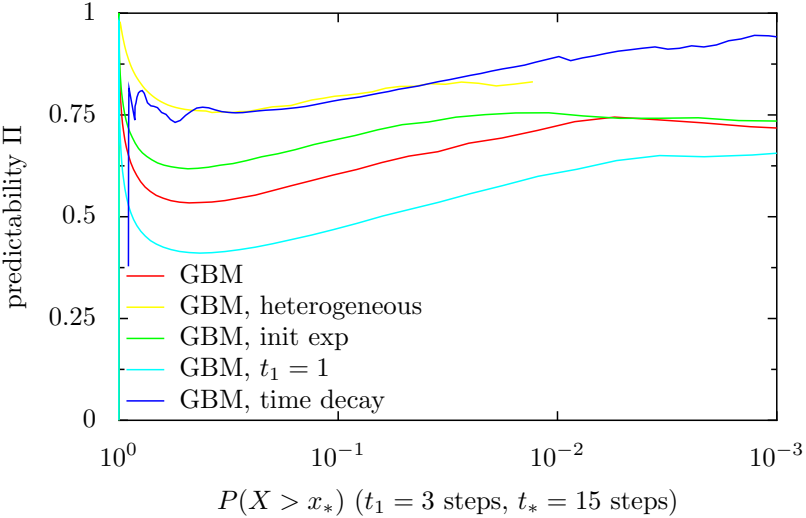

Supplement: Figure S2 — Predictability of simple stochastic processes. An ensemble of random walkers evolve through the dynamics , where (Geometric Brownian Motion with Gaussian steps. The predictability of extreme events was computed for steps and steps. GBM: and ; GBM heterogeneous: and , fixed in time; GBM, init exp: and ; GBM, the same as GBM for ; GBM, time decay: model proposed in Ref. [15], similar to GBM heterogeneous but with a rate that decays in time ( with ; is a log-normal surviving probability with parameters and ). (PDF) [file pone.0111506.s002.pdf]
