# Supplementary material for: Predictability of Extreme Events in Social Media
Source: PLoS One. 2014 Nov 4;9(11):e111506. doi: 10.1371/journal.pone.0111506 (PMC4219754; doi:10.1371/journal.pone.0111506)
Supplement: Appendix S1 — Details on procedures, analysis and data. (PDF) [file pone.0111506.s003.pdf]

Predictability of extreme events in social media
Supporting Information: Appendix S1

José M. Miotto, Eduardo G. Altmann

Contents

1 Datasets **1**
1.1 YouTube . . . 1
1.2 Usenet . . . 1
1.3 Stack-Overflow . . . 1
1.4 PLOS ONE . . . 2

2 Extreme Value statistics **2**
2.1 Overall distributions . . . 2
2.2 YouTube . . . 2
2.3 Usenet . . . 3
2.4 Stack Overflow . . . 3
2.5 PLOS ONE . . . 3

3 Quality of binary predictions **4**

4 Demonstration that strategy LD (Bayes classifier) is dominant **4**

5 Computation of Π for the optimal strategy **4**

1 Datasets

We worked with four different datasets. Each of them is composed by a collection of items wich have an associated time series of activity that we consider a proxy of attention; in the case of Stack-Overflow and Usenet, each post or vote has its own timestamp, and in the case of YouTube and PLOS all the views are collected in time bins (day and month, respectively). All the data is available at Ref. [1] in a format prepared to be used with a Python script (also included in the fileset) to compute the Predictability measure we propose.

In Fig. S1 we show the distributions of activity at a given time t_*.

1.1 YouTube

The YouTube dataset is an unbiased collection (16.2 million) of publicly available videos published between Jan. 2012 and Apr. 2013. The data was retrieved through the YouTube API provided by Google (`https://developers.google.com/youtube/code`).

1.2 Usenet

The Usenet dataset is a collection (0.8 million) of publicly available threads in online discussion groups (they can be retrieved directly through Google Groups, which holds a large archive of them; for example, to view the group `comp.os.linux`, visit `https://groups.google.com/forum/#!forum/comp.os.linux`). All posts published in the groups indicated in Sec. SI 2.3 were retrieved before March 2008, see Ref. [2] for details.

1.3 Stack-Overflow

The Stack-Overflow dataset is a collection (4.6 million) of all publicly available questions in the website stack-overflow.com, posted between Jul. 2008 and Mar. 2013. The data used is the dump of the website released by the website in Mar. 2013 (`http://archive.org/details/stackexchange`).

1.4 PLOS ONE

The PLOS ONE dataset is a collection (72246) of publicly available scientific publications of the journal PLOS ONE, between Dec. 2006 and Aug. 2013. The data was retrieved through the API provided by PLOS (`http://api.plos.org`), but is identical to the one published in Ref. [3].

2 Extreme Value statistics

The cumulative Generalized Pareto Distribution is given by [4]

$$F(x) = 1 - \left(1 + \frac{x - x_p}{\sigma\alpha}\right)^{-\alpha} \tag{1}$$

for $x > x_p$. In our analysis it is essential to consider the discretization of the observations (specially for small values), and therefore we used a discretized version of the Generalized Pareto Distribution, which has probability density function

$$f(x) = \frac{(\sigma\alpha)^{-(\alpha+1)}}{\zeta(\alpha+1, \sigma\alpha)} \left(1 + \frac{x - x_p}{\sigma\alpha}\right)^{-(\alpha+1)} \tag{2}$$

where ζ is the Hurwitz Zeta function.

The parameters of Eq. (2) are estimated using maximum likelihood fit [5]. The goodness of the fit is measured by the p-value computed as the probability of having a Kolmogorov-Smirnov statistic, between randomly sampled data (from the fitted distribution) and their fit, bigger than the one measured between the real data and their fit [6]. Fits with p-values bigger than 0.05 are regarded as acceptable.

For each database we considered the fit of all data (see SI Sec. 2.1) and of the data partitioned in groups as described below. For the case of the partitioned data, we report the results for the lowest threshold that guarantees statistical significance for at least 80% of the groups analyzed for each database. This allows us to have a good estimation of the AUC for a wide range of x_* values (see Fig. 3).

2.1 Overall distributions

Here we report the fits to the whole databases.

Database	α	σ	x_p	KS	p	N_{fit}	N
YouTube	0.69	55.3	20	0.003	0.00	7621233	13284409
Usenet	1.56	5.15	1	0.028	0.96	344608	871931
Stack-Overflow	2.03	7.7	15	0.027	0.38	12193	4397194
PLOS ONE	1.41	1083.22	2300	0.009	0.14	5948	72244

2.2 YouTube

The property used to partition the dataset was the *Category*: each uploaded video belongs to a pre-defined Category selected by the user (15 in total). The threshold used in the reported results is $x_p = 20$.

Group	α	σ	KS	p	N_{fit}	$P(g)$
Autos & Vehicles	0.75	62.9	0.005	0.78	711022	7.4%
Comedy	0.84	55.2	0.005	0.85	571719	8.3%
Education	0.61	45.3	0.009	0.07	502299	7.0%
Entertainment	0.88	46.2	0.007	0.54	494318	7.7%
Film & Animation	0.64	63.5	0.006	0.81	375084	5.1%
Gaming	0.58	74.8	0.009	0.12	575361	6.3%
Howto & Style	0.69	48.8	0.006	0.81	445804	6.4%
Music	0.70	59.3	0.006	0.84	530635	6.4%
News	0.50	51.7	0.017	0.02	232925	2.9%
Nonprofits & Activism	0.92	40.4	0.004	1.00	428223	7.3%
People & Blogs	0.97	33.8	0.007	0.57	476004	7.6%
Pets & Animals	0.71	101.1	0.017	0.00	752916	8.2%
Science & Technology	0.65	76.8	0.012	0.01	655954	7.1%
Sports	0.86	53.5	0.005	0.94	479078	6.6%
Travel & Events	0.70	43.8	0.005	0.99	389891	5.6%

2.3 Usenet

The dataset is partitioned in 9 different *Discussion Groups*, which cover different topics. The threshold used is $x_p = 1$.

Group	α	σ	KS	p	N_{fit}	$P(g)$
alt.rap	4.36	4.6	0.046	0.25	23112	7.3%
comp.os.linux.misc	3.60	3.4	0.061	0.00	69099	18.6%
rec.arts.poems	1.67	4.0	0.028	1.00	42199	23.5%
rec.arts.sf.written	1.15	8.2	0.020	0.23	39040	9.1%
rec.music.classical	2.03	5.6	0.030	0.60	36151	10.0%
rec.music.country.western	2.02	4.9	0.032	0.84	29131	8.6%
rec.music.hip-hop	3.36	5.8	0.033	0.83	48792	12.1%
sci.physics.fusion	2.08	4.5	0.033	0.89	4622	1.7%
talk.origins	1.63	12.7	0.017	0.10	52454	9.1%

2.4 Stack Overflow

The Stack Overflow dataset is composed by questions and answers of computer-science topics. We use the questions and divide them in groups g according to their tags. Since each question has many tags a classification procedure was performed. Programming languages are the most common type of tag and therefore we selected the 10 more common tags of this type (see list in the table below). Tags which contained one of these 10 tags as a substring were considered as equivalent to the short tags. Similarly, tags that could be associated to a single programing language were also merged. The remaining tags were grouped in a group labelled *rest*, which included also all cases in the intersection of two or more programming language groups. The complete grouping of the tags can be seen at Ref. [1] in the file "Stack-Overflow Lemmas". The threshold used for the fits is $x_p = 15$.

Group	α	σ	KS	p	N_{fit}	$P(g)$
.net	2.06	7.4	0.039	0.74	1802	12.2%
c	1.54	8.0	0.030	1.00	348	1.5%
c++	1.82	7.1	0.036	0.94	1125	3.5%
css	2.35	7.9	0.049	1.00	163	1.4%
html	1.86	6.5	0.050	1.00	114	1.0%
java	1.87	7.8	0.045	0.81	909	7.6%
javascript	1.83	7.9	0.031	0.99	747	8.6%
php	2.10	7.9	0.040	0.99	293	5.6%
python	2.11	6.5	0.055	0.91	516	4.1%
rest	2.14	8.0	0.029	0.76	5969	49.7%
sql	3.14	8.9	0.054	0.97	207	4.8%

2.5 PLOS ONE

For the PLOS ONE dataset, we chose the amount of authors papers have as the grouping factor; the group labelled 12, actually contains all the papers with more than 11 authors. The threshold used for the fits is $x_p = 1400$.

Group	α	σ	KS	p	N_{fit}	$P(g)$
1	1.64	1262.7	0.043	0.46	187	1.1%
2	1.27	897.9	0.015	0.82	941	6.7%
3	1.61	904.5	0.016	0.44	1393	10.5%
4	1.47	686.5	0.009	0.97	1498	12.2%
5	1.52	723.4	0.019	0.10	1565	12.6%
6	1.59	672.3	0.014	0.66	1313	11.4%
7	1.50	682.6	0.018	0.49	1142	10.3%
8	1.65	643.5	0.023	0.13	931	8.6%
9	1.85	666.9	0.034	0.03	794	6.8%
10	1.60	642.7	0.033	0.07	587	5.3%
11	1.74	693.8	0.040	0.04	474	3.9%
12+	1.88	767.1	0.020	0.08	1665	10.6%

3 Quality of binary predictions

Comparing binary predictions and observations gives four possible results, given by the combination of the prediction (positive or negative) and its success (true or false). If A denotes the prediction of an event (an alarm), the hit rate (or True Positive Rate) and the false alarm rate (or False Positive Rate) are defined as

$$\begin{aligned} \text{hit rate} &\equiv \frac{\text{number of true positives}}{\text{number of positives}} = P(A|E), \\ \text{false alarm rate} &\equiv \frac{\text{number of false positives}}{\text{number of negatives}} = P(A|\bar{E}). \end{aligned} \tag{3}$$

These are analogous to measures like Accuracy and Specificity or Precision and Recall. Prediction strategies typically have a specificity parameter (e.g., controlling the rate of false positives). Varying this parameter, a prediction curve that goes from $(0,0)$ to $(1,1)$ is built in the hit$\times$false-alarm space.

4 Demonstration that strategy LD (Bayes classifier) is dominant

A strategy is dominant when for any given false alarm rate, the hit rate is maximized. Following definition (3), we write the x and y coordinates of the hit$\times$false-alarm plot as

$$\begin{aligned} \text{hit rate} &\equiv P(A|E) = \textstyle\sum_{g=1}^{G} P(A|g)P(g|E) = \sum_{g=1}^{G} \pi_g y_g \equiv y, \\ \text{false-alarm rate} &\equiv P(A|\bar{E}) = \textstyle\sum_{g=1}^{G} P(A|g)P(g|\bar{E}) = \sum_{g=1}^{G} \pi_g x_g \equiv x, \end{aligned} \tag{4}$$

where for notational convenience $y_g \equiv P(g|E)$, $x_g \equiv P(g|\bar{E})$, and $\pi_g \equiv P(A|g)$. Since predictions are issued based only on the information about the groups, strategies (both deterministic and stochastic) are defined uniquely by π_g, while x_g and y_g are estimated from data. The computation of the dominant strategy corresponds to finding the π_g's that maximize y with the constraint $\sum_{g=1}^{G} \pi_g x_g = x$. This problem can be solved exactly by applying the simplex method. Define h such that $\sum_{g<h} x_g < x < \sum_{g\leq h} x_g$; we write Eq. (4) as:

$$\begin{aligned} y - \sum_{g<h} y_g &= -\sum_{g<h}(1-\pi_g)y_g + \sum_{g>h} \pi_g y_g + \pi_h y_h, \\ x - \sum_{g<h} x_g &= -\sum_{g<h}(1-\pi_g)x_g + \sum_{g>h} \pi_g x_g + \pi_h x_h. \end{aligned} \tag{5}$$

Isolating π_h in the lower equation and introducing it in the top one we obtain

$$y = \sum_{g<h} y_g + x\frac{y_h}{x_h} \tag{6}$$

$$-\sum_{g<h}(1-\pi_g)x_g\left(\frac{y_g}{x_g} - \frac{y_h}{x_h}\right) + \sum_{g>h} \pi_g x_g \left(\frac{y_g}{x_g} - \frac{y_h}{x_h}\right). \tag{7}$$

Notice that y_g/x_g is the contribution of the group g to the slope of the prediction curve in the hit$\times$false-alarm space. If the G groups are ordered by decreasing $P(E|g)$, then y_g/x_g also decreases with g. Therefore $(y_g/x_g - y_h/x_h) > 0$ for $g < h$ and $(y_g/x_g - y_h/x_h) > 0$ for $g > h$ and Eq. (6) is maximized by choosing π_g such that the two last terms vanish. This is achieved choosing

$$\pi_g = \begin{cases} 1 & g < h, \\ \frac{x - \sum_{g<h} x_g}{x_h} & g = h, \\ 0 & g > h, \end{cases} \tag{8}$$

which correspond to issuing positive predictions only to the h groups with largest $P(E|g)$ and is equivalent to strategy (LD) mentioned in the main text. Positive events are predicted for the group h in Eq. (8) as much as needed to reach the required false positive rate x.

5 Computation of Π for the optimal strategy

As illustrated in Fig. 2(b), the partition performed by the optimal strategy defines G different intervals in the hit and false alarm axis (the points for which $P(E|g) = P_*$, $g \in \{1 \ldots G\}$) and therefore G^2 rectangles in the

hit×false-alarm space. The (g,h) rectangle has height $P(h)P(E|h)/P(E) = P(h|E)$, width $P(g|\bar{E})$ (where $\bar{E}$ is the complement of E, i.e., $P(\bar{E}|g) = 1 - P(E|g)$), and therefore it has an area $A_{g,h} = P(h|E)P(g|\bar{E})$. The curve of strategy (LD) is the union of the diagonals of the $g = h$ rectangles (which are obtained by increasing p_*). Π is two times the sum of the rectangles and triangles under this curve minus half of all the area:

$$\begin{aligned}
\Pi &= 2\left[\sum_g \sum_{h<g} A_{g,h} + \frac{1}{2}\sum_g A_{g,g} - \frac{1}{2}\sum_g \sum_h A_{g,h}\right] \\
&= \sum_g \sum_{h<g} A_{g,h} - \sum_g \sum_{h>g} A_{g,h} \\
&= \sum_g \sum_{h<g} (A_{g,h} - A_{h,g}) \\
&= \sum_g \sum_{h<g} P(h|E)P(g|\bar{E}) - P(h|\bar{E})P(g|E) \\
&= \frac{\sum_g \sum_{h<g} P(g)P(h)\left(P(E|h) - P(E|g)\right)}{P(E)(1-P(E))},
\end{aligned} \tag{9}$$

where we used $\sum_g \sum_h A_{g,h} = 1$. This finishes our demonstration of Eq. 2.

References

[1] Miotto JM, Altmann EG (2014). Time series of social media activity: Youtube, usenet, stack-overflow. Available: `http://dx.doi.org/10.6084/m9.figshare.1160515`. Accessed 2014 Oct 7.

[2] Altmann EG, Pierrehumbert JB, Motter A (2011) Niche as a determinant of word fate in online groups. PLoS ONE 6.

[3] Fenner M, Lin J (2013). Cumulative usage statistics for plos papers from plos website. Available: `http://dx.doi.org/10.6084/m9.figshare.816962`. Accessed 2014 Jun 4.

[4] Coles S (2001) An introduction to statistical modeling of extreme values. Springer.

[5] Grimshaw SD (1993) Computing maximum likelihood estimates for the generalized pareto distribution. Technometrics 35: 185–191.

[6] Clauset A, Shalizi CR, Newman MEJ (2009) Power-law distributions in empirical data. SIAM Rev 51: 661–703.
